# Whey Protein Enzymatic Breakdown: Synthesis, Analysis, and Discovery of New Biologically Active Peptides in Papain-Derived Hydrolysates

**DOI:** 10.3390/molecules30071451

**Published:** 2025-03-25

**Authors:** Michał Czelej, Katarzyna Garbacz, Tomasz Czernecki, Kamila Rachwał, Jacek Wawrzykowski, Adam Waśko

**Affiliations:** 1 Biolive Innovation Sp. z.o.o., Dobrzańskiego 3, 20-262 Lublin, Polandkatarzyna.garbacz@up.lublin.pl (K.G.); 2 Department of Biotechnology, Microbiology and Human Nutrition, Faculty of Food Science and Biotechnology, University of Life Sciences in Lublin, Skromna 8, 20-704 Lublin, Poland; tomasz.czernecki@up.lublin.pl (T.C.); adam.wasko@up.lublin.pl (A.W.); 3 Department of Biochemistry, Faculty of Veterinary Medicine, University of Life Sciences in Lublin, Akademicka 12, 20-033 Lublin, Poland

**Keywords:** enzymatic hydrolysis, whey protein, biological activity of peptides, LC-MS identification of peptides

## Abstract

Bioactive peptides derived from milk proteins offer promising potential that can be unlocked through hydrolysis. Enzymatic hydrolysis is particularly noteworthy because of its mild conditions and its efficacy in producing peptides with various biological activities. This study focused on creating whey protein hydrolysates using three enzymes: pepsin, trypsin, and papain. The degree of hydrolysis and the antioxidant properties of the resulting peptides were evaluated, and papain demonstrated the highest degree of hydrolysis, leading to its selection for further investigation. LC-MS was employed to identify peptide sequences from the papain-derived hydrolysate, resulting in the identification of 107 distinct peptide sequences These peptides were predicted to exhibit a range of potential biological activities, including antihypertensive, antidiabetic, antioxidant, antimicrobial, and immunomodulatory effects, as well as roles in regulating glucose homeostasis, maintaining cardiovascular health, and supporting overall metabolic function. In vitro tests revealed the significant antioxidant and antibacterial properties of the hydrolysate, confirming the potential of papain-derived peptides for use in functional food and pharmaceutical applications. The novelty of this study lies in the identification of novel peptides with promising biological activities. Additional in vitro and in vivo studies are required to fully elucidate the health benefits of these peptides.

## 1. Introduction

Whey, a protein-rich fraction obtained during the acid or enzymatic processing of dairy products in cheese or casein production, is frequently considered a by-product, constituting approximately 85–90% of the milk volume. However, because of its high nutritional value and health-promoting properties, whey can be used for the production of dietary supplements and nutraceuticals [1,2]. This co-product contains various soluble milk components, including proteins, lipids, lactose, polyphenols, minerals, and vitamins [3]. Among the most prevalent proteins in whey are β-lactoglobulin, α-lactalbumin, bovine serum albumin, and immunoglobulins, with lactoferrin, lactoperoxidase, and proteose peptone present in lower quantities. Whey proteins also include glycomacropeptides released during plasmin-mediated hydrolysis, which are exclusively present in sweet whey [4].

Whey proteins are widely acknowledged for their unique composition and functional properties that render them valuable in the food industry. These proteins exhibit high solubility and are susceptible to heat denaturation owing to their globular conformation because of their highly diluted ingredients and perishability [2]. Consequently, various processing methods have been developed to enhance the usability of whey, with one of the most promising being hydrolysis, which produces peptides with potential biological activities [5].

Hydrolysis is crucial for unlocking the bioactive potential of whey proteins. It can be performed through chemical or enzymatic means as well as through fermentation or physical processes. Despite its cost-effectiveness and simplicity, chemical hydrolysis presents several drawbacks, including the potential for toxic residues and diminished nutritional value due to harsh processing conditions [6]. In contrast, enzymatic hydrolysis offers more favourable conditions, allowing the controlled release of peptides with specific biological activities [3,7]. The principal enzymes utilised include gastrointestinal enzymes (pepsin, pancreatin, trypsin, chymotrypsin), plant-derived enzymes (e.g., papain, bromelain, actinidin), and proteolytic microorganisms or microbial proteases (e.g., proteases from lactic acid bacteria and flavourzyme from *Aspergillus oryzae*) [6,8]. Despite the known benefits of enzymatic hydrolysis, the specific bioactive potential of papain-derived whey protein peptides, particularly in terms of their antioxidant and antimicrobial activities, remains underexplored. Additionally, the fermentation process utilises the proteolytic activity of microorganisms, whereas methods such as microwaves or ultrasound can also facilitate peptide production [9].

Different enzymatic digestion processes yield different quantities and types of peptides in the same food matrix. The selection of a particular enzyme to catalyse protein hydrolysis is guided by the evaluation of its selectivity and available literature data, indicating its applicability. Enzymatic hydrolysis of whey proteins employs various proteases, each influencing the composition and bioactivity of the resulting peptides. For example, Alcalase, a serine protease, efficiently generates antioxidant and ACE-inhibitory peptides, while trypsin and chymotrypsin, often used together, enhance the release of bioactive sequences [10,11,12]. Pepsin hydrolysis improves antioxidant and antihypertensive properties, whereas plant-derived enzymes such as papain, bromelain, and ficin contribute to antihypertensive peptide production [12,13]. Microbial proteases (Protease S and M) yield hydrolysates with a high degree of hydrolysis and significant bioactive properties [14,15]. The functional properties of peptides derived from whey proteins are influenced by their amino acid composition. For instance, peptides with aromatic and nonpolar residues tend to exhibit enhanced antioxidant activity, while cationic and aromatic sequences are associated with ACE inhibition. Additionally, other peptides may demonstrate antimicrobial, immunomodulatory, antidiabetic, or opioid-like activities [3,12,16].

In this study, the most commonly used gastrointestinal enzymes, pepsin and trypsin, were used for hydrolysis. Additionally, they were selected based on literature data suggesting that they are capable of generating peptides with dipeptidyl peptidase IV (DPPIV) and angiotensin-converting enzyme (ACE) inhibitory [17,18], bactericidal [19], antihypertensive, antioxidant [20], and cytoprotective [21] properties. In recent years, research has been conducted to obtain bioactive whey protein peptides using enzymes of plant origin. Previously, it was reported that plant-derived proteases (cardosins A and B, papain, bromelain, and ficin) could be utilised to obtain peptides with ACE inhibitory activity [13,22].

The enzymatic hydrolysis of whey proteins enhances their nutritional and functional properties, rendering them suitable for a diverse range of applications in the food and health industries. This process generates bioactive peptides with improved digestibility, solubility, and physiological activities, further increasing their potential benefits. Hydrolysis augments the nutritional value of whey proteins by cleaving them into smaller peptides and amino acids, which are more readily digested and absorbed. This process substantially reduces the antigenic properties of whey proteins, making them suitable for use in hypoallergenic food products [16]. Regarding effects on functional characteristics, enzymatic hydrolysis of whey proteins improves solubility across a wide pH range, promotes viscosity through water binding, and facilitates cohesion, adhesion, and elasticity [23]. Enzymatic hydrolysis can also enhance the foaming and emulsifying properties of whey proteins, rendering them suitable for use in sports nutrition and as stabilisers in acidic beverages [24].

Enzymatic hydrolysis is utilised to generate peptides with a diverse range of bioactive properties, which have applications in the development of specialised foods and beverages for sports nutrition and preventive nutrition for individuals with allergies to bovine milk proteins [25]. The selection of enzymes and hydrolysis conditions plays a crucial role in determining the specific benefits and applications of the resulting hydrolysates. It determines the degree of protein hydrolysis (DH), which influences the functional properties of the hydrolysates. Higher DH is also frequently correlated with increased bioactivity [11]. Consequently, different enzymes employed in hydrolysis can produce distinct peptide profiles with varying bioactivity [13,26].

Bioactive peptides released from whey proteins during hydrolysis exhibit a broad spectrum of health-promoting properties, including antimicrobial, antidiabetic, antioxidant, and immunomodulatory effects. These properties have generated significant interest in food and nutraceutical industries, particularly in the development of functional foods and supplements. Several commercially available products incorporate bioactive whey peptides for specific therapeutic purposes, such as antihypertensive, anti-inflammatory, and sleep-inducing effects [6]. Additionally, whey peptides may possess anticancer, cytoprotective, mineral-chelating, osteoanabolic, hypocholesterolemic, opioid, and antithrombotic properties. However, the market for functional foods enriched with whey-derived bioactive peptides is relatively underdeveloped, highlighting the need for further research in this area. The discovery of novel peptide sequences with specific biological properties may influence the development of innovative products enriched with health functions such as antioxidant activity, anticancer properties, or improvement of the gut microbiota.

The use of bioactive peptides in functional foods and supplements represents a highly pertinent and expanding field of research. Given the potential of whey as a rich source of such peptides, there is significant demand for efficient peptide production and characterisation methods. Owing to their health-promoting properties, these peptides hold considerable promise as ingredients in fortified foods, offering enhanced nutritional benefits. However, there is a paucity of reports in the literature regarding the activity of peptides generated from whey proteins using papain, and studies have not identified the specific sequences of peptides obtained using this method. This study aimed to evaluate and compare the hydrolysis efficiency and bioactive potential of whey peptides generated using pepsin, papain, and trypsin. Particular emphasis was placed on the identification of novel peptide sequences in papain hydrolysate and the assessment of their potential biological activity. This study employed a comprehensive approach that integrated experimental analysis of biological activities (antioxidant and antimicrobial), LC-MS analysis of the peptide composition obtained during papain hydrolysis, and in silico prediction of their activities. Owing to the acquisition and identification of new peptides, these findings may contribute to the development of novel food products, nutraceuticals, or therapeutic agents, supporting the growing interest in bioactive peptides for health promotion. This study aimed to evaluate the efficiency of papain in hydrolysing whey proteins, identifying novel bioactive peptides, and assessing their potential antioxidant and antimicrobial activities.

## 2. Results and Discussion

### 2.1. Degree of Hydrolysis (DH) of Whey Proteins by Various Enzymes

**Figure 1:**
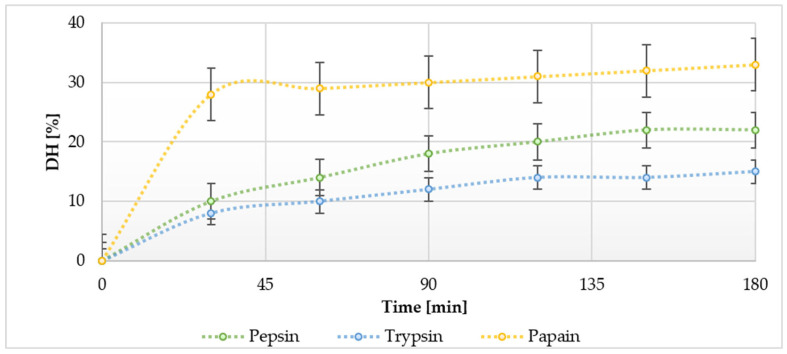
Degree of hydrolysis (%) of whey proteins by pepsin, trypsin, and papain at different time points.

Enzyme activity plays a crucial role in protein hydrolysis; therefore, in the initial phase of the study, enzymatic hydrolysis of whey proteins was conducted using three distinct enzymes under identical reaction conditions (selected following preliminary optimisation). Various commercial proteases, including whey proteins, have been successfully evaluated for the production of bioactive hydrolysates from milk. In this study, reactions catalysed by trypsin, pepsin, and papain were selected for analysis. For each variant, the degree of hydrolysis was determined at different time intervals. Figure 1 illustrates the effects of enzyme specificity and reaction time on the degree of hydrolysis (DH). Among the three enzymes employed in this study, whey proteins hydrolysed with papain after a 3 h reaction exhibited significantly higher DH values than whey hydrolysed with trypsin and pepsin. The hydrolysis rates for trypsin, pepsin, and papain were 15 ± 1.6%, 22 ± 2.8%, and 33 ± 3.7%, respectively (Figure 1). Consequently, the highest efficiency of the enzymatic reaction, assessed by the ratio of released peptides to total protein content, was observed when papain hydrolysis was employed (DH 33 ± 3.7% after 3 h). It is also noteworthy that a reaction duration of merely half an hour facilitated a high degree of whey protein hydrolysis.

Papain is an enzyme known for its advantages, including faster rates and the ability to operate under mild conditions. This unique property helps preserve the bioactivity of the released peptides, which is often compromised by harsher enzymatic or chemical hydrolysis processes, while also enabling a reduction in production costs and improving yield and productivity [13]. In addition, papain has a narrow pH range (5–7), but its activity is advantageous over a broad temperature range, including higher temperatures (<90 °C) [27].

Studies on whey hydrolysis have demonstrated different levels of hydrolysis for the different enzymes utilised. This is because the efficiency of enzymatic hydrolysis of whey proteins depends on factors such as the type of enzyme and substrate, their ratio, hydrolysis conditions, and mixing conditions [28]. For example, in a study conducted by Wang et al. [19], whey protein hydrolysis was performed using three different enzymes. A significantly lower degree of hydrolysis was observed in variants with 3 h hydrolysis with papain (DH 9.3 ± 0.3%), whereas superior results were obtained for samples hydrolysed with alkalase (6 h, DH 32.2 ± 2.5%) and trypsin (4 h, DH 25.2 ± 1.7%). However, the reaction times and temperatures applied were different. Additionally, Kaur et al. [27] observed lower levels of whey protein hydrolysis using papain (DH of 17.69%), while results very similar to those obtained in this work (DH of 32.28%) were demonstrated by Du et al. [29] in their study.

### 2.2. Antioxidant Properties of Peptides Obtained via Enzymatic Hydrolysis of Whey Proteins

In the subsequent phase, the antioxidant properties of whey hydrolysates obtained via enzymatic hydrolysis using pepsin, trypsin, and papain were determined (Table 1). Given the increasing prevalence of civilisation diseases, interest in the antioxidant properties of food products has recently intensified. Antioxidant properties pertain to the capacity of certain molecules to neutralise excessive amounts of free oxygen radicals in the body, which are responsible for cellular damage and associated with the occurrence of diseases. Two methods were employed to determine antioxidant activity: the reduction of metal ions to lower-oxidation ions by the antioxidant under investigation (FRAP) and the ability to scavenge stable free radicals (ABTS+ and DPPH). The analyses conducted in this study showed the significant antioxidant potential of all hydrolysates obtained (Table 1).

Assessment of ABTS+ cation radical scavenging activity is the predominant method used to quantify the antioxidant activity of food ingredients. Among the hydrolysates obtained in the 3 h reaction with various enzymes, the highest activity was observed for whey proteins digested with papain (527.36 ± 25.13 (eq Trolox µmol/g)), followed by trypsin and pepsin (361.96 ± 31.02 and 297.44 ± 24.11 (eq Trolox µmol/g), respectively).

Owing to its simplicity, short analysis time, and relatively high accuracy and reproducibility of results, an assay based on testing the reactivity of the DPPH (1,1-diphenyl-2-picrylhydrazyl) radical with the sample is one of the most widely utilised methods for measuring the scavenging potential of peptides. It is used to measure the total antioxidant potential of various food products. The results of the DPPH assay of whey hydrolysates extracted with different enzymes revealed the highest free radical scavenging potential of the formula obtained using trypsin (19.68 ± 1.5 (eq Trolox µmol/g)). This capacity using papain and pepsin was marginally lower (17.56 ± 2.16 and 16.22 ± 2.03 (eq Trolox µmol/g), respectively).

The FRAP method quantifies antioxidant capacity based on the influence of antioxidants present in the sample on the rate of reduction of the ferric iron (III)/ferricyanide complex to ferrous iron (II). In this study, the assay demonstrated the highest reducing potential in the whey hydrolysate prepared using pepsin catalysis (4.87 ± 0.22 (eq Trolox µmol/g)), whilst a marginally lower value was observed for the hydrolysate prepared using papain (3.56 ± 0.17 (eq Trolox µmol/g)). The hydrolysate obtained using trypsin exhibited the lowest antioxidant potential (1.75 ± 0.03 (eq Trolox µmol/g)).

The differences observed in the free-radical-reducing activity of whey hydrolysates prepared using different enzymes may be attributed to the specificity of the enzyme for a particular site in the peptide chain. This phenomenon is also associated with the degree of hydrolysis (DH) of the constituent proteins, which ultimately results in hydrolysates containing different amino acid compositions [30]. DH and the enzymes utilised to catalyse hydrolysis play a crucial role in determining the reducing power of protein hydrolysates. The observed differences in antioxidant activity among the tested protein hydrolysates can be associated with the specific amino acid profiles of the peptides [31]. The amino acid sequence also exerts a significant influence on the potency of the antioxidant activity of peptides owing to variations in the activity of individual amino acids related to differences in the side chain structure. This activity is attributed to readily oxidised groups: the indole group in tryptophan, imidazole group in histidine, thiol in cysteine, phenolic hydroxyl group in tyrosine, and thioether in methionine [32]. Ohashi et al. [33] demonstrated that tyrosine-containing tripeptides exhibited higher antioxidant activity against the hydrophilic radical ABTS than histidine-containing tripeptides, suggesting an essential role for tryptophan and tyrosine residues in the determination of radical scavenging activity. The tripeptides examined showed weak scavenging activity against the hydrophobic DPPH radical, and their antioxidant activity against linoleic acid peroxidation demonstrated a high correlation with ABTS assays and a low correlation with FRAP and DPPH assays [33]. This explains the differences in the antioxidant potential assay results observed in this study.

### 2.3. Antibacterial Activity of Whey Peptides

At this stage of the investigation, only whey hydrolysate produced using papain was analysed. This hydrolysate was selected based on its superior degree of hydrolysis and potent antioxidant activity (Table 1) [34,35].

The effects of whey peptides on the growth of eight bacterial isolates obtained from spoilt food were investigated. Growth inhibition zones were used as a measure of the antimicrobial activity of whey hydrolysates. Growth inhibition zones surrounding the wells containing whey-derived peptides were observed for four bacterial strains tested (*Bacillus* sp., *Bacillus megaterium*, *Staphylococcus capitis*, and *Rhizobium radiobacter*) (Table 2). The highest antibacterial activity was observed against Bacillus sp. with an inhibition zone of 11 mm. No growth inhibition was detected in *B. cereus*, *B. pumilus*, *E. aurantiacum*, or *B. diminut*. These results indicate a selective inhibitory effect of the peptides contained in whey hydrolysates on the growth of certain bacterial strains. The observed effect can be attributed to the capacity of bacteria, including *Bacillus* spp., to secrete extracellular proteases capable of inactivating antibacterial peptides [36].

Numerous previous studies have demonstrated that enzymatic hydrolysis of whey proteins results in the release of antimicrobial peptides, which are encrypted in whey proteins. The positive effect of papain enzymatic hydrolysis on antimicrobial activity has previously been demonstrated for papain hydrolysates of buffalo and camel milk whey proteins [37,38,39].

Peptides derived from β-lactoglobulin degradation, including the AASDISLLDAQSAPLR peptide identified in this study, were responsible for the observed antimicrobial activity of the hydrolysate. This effect may also be attributable to the presence of other sequences with antimicrobial activity in the obtained peptides derived from the hydrolysis of β-lactoglobulin, namely VAGTWY, VLVLDTDYK, and IPAVFK, which were previously identified by Pellegrini et al. [40]. The presence of antimicrobial peptides in the hydrolysate suggests that the addition of these peptides to food products may exert an inhibitory effect on the growth of certain undesirable bacteria present in food.

### 2.4. Identification of Peptides Derived from Whey and Their Predicted Biological Activity

Following the hydrolysis methodology involving papain and the aforementioned conditions, 107 peptides were extracted and identified. LC-MS analysis revealed peptides of varying lengths ranging from 7 to 27 amino acids. A comprehensive list of all identified peptide sequences, including information on the proteins from which they were derived and the biological activities of the peptides, as determined from data available in databases and predicted from the sequences, is presented in Table S1.

The molecular weights of peptides obtained from the hydrolysis of whey proteins are contingent on the enzymatic reaction conditions employed, particularly the enzyme type utilised. The bioactivity of the resultant hydrolysate was determined based on its final composition and amino acid sequence. In this study, multiple predicted biological activities were attributed to each peptide. It is not uncommon for a single peptide to exhibit multiple functions because of the interconnectedness of metabolic pathways that govern specific functions. Furthermore, certain peptides may function as signalling molecules, thereby exerting systemic effects.

Based on peptide sequences obtained using LC-MS, the biological activities of several peptides were identified using a database search (BIOPEP-UWM database). These include zinc-binding peptides, such as VEELKPTPEGDLEIL and ELKPTPEGDLEIL, derived from β-lactoglobulin digestion. Previous studies have demonstrated that peptides interact with zinc ions to form chemically stable and soluble chelates. These chelates are involved in elemental transport and can prevent zinc from interacting with various compounds in the gastrointestinal tract, such as phytic acid. Zinc peptide chelates can be utilised as a novel route for supplementing the human body with zinc, as the zinc present in such a chelate can enter the body via the peptide absorption mode [41]. Such zinc-binding peptides may have valuable nutritional applications as dietary zinc carriers to enhance the bioavailability and bioaccessibility of this element. Furthermore, peptides possessing zinc ligands are pharmacologically relevant as zinc-dependent enzyme inhibitors and are potentially efficacious in controlling inflammatory diseases [42].

The APFPEVF peptide derived from α-S1-casein digestion was identified. It has been recognised for its potential application in the prevention and treatment of pathological conditions associated with alpha-glucosidase [43,44]. The databases also provided information on the activity of peptides obtained during the hydrolysis of whey using β-casein, namely VYPFPGPIH, LVYPFPGPIH, and YPFPGPIPNS. The VYPFPGPIH peptide has been reported to inhibit prolyl endopeptidase (PEP) activity, which has been associated with neurodegenerative disorders.

PEP inhibitors are considered capable of reversing memory loss induced by amnesic compounds [45]. LVYPFPGPIH has been identified as an ACE inhibitor that exhibits antihypertensive effects, whereas YPFPGPIPNS is known as β-casomorphin-11, which is an opioid receptor agonist. Bioactive peptides from this family are capable of binding to and activating opioid receptors located in various regions of the body (including the immune system, gastrointestinal tract, and potentially the central nervous system) and may differentially affect processes such as immunosuppression, gastrointestinal nutrient absorption, opioid signalling, hyperglycaemia, and oxidative stress [46,47].

Analysis of the predicted activity of other peptides indicated that they may exert a variety of beneficial physiological effects, including reducing blood pressure, preventing cardiovascular disease, exhibiting hypolipidaemic effects, maintaining glucose homeostasis, regulating stomach mucosal membrane activity, influencing the nervous system, and functioning as antidiabetic, anticancer, anti-inflammatory, immunomodulatory, antimicrobial, antioxidant, and osteoanabolic agents.

Previous studies have demonstrated that the milk proteome comprises peptides that are inactive in the native protein, but upon release, constitute biologically active peptides capable of interacting with relevant receptors and regulating physiological functions in the body. These peptides have diverse biological activities. Peptides released from whey proteins have demonstrated antihypertensive (ACE inhibitors), antioxidant, anticancer, cognitive, and memory-enhancing effects [13].

Antioxidant and antimicrobial activities are considered to be the primary common characteristics of biologically active peptides. In the present study, 16 peptides potentially responsible for the observed antimicrobial activity of the hydrolysates were identified. In contrast, 58 peptides derived from whey digestion were potentially associated with in vitro antioxidant activity. Antioxidant activity is also associated with cancer prevention. Among the identified peptides, 12 exhibited potential anticancer activity. Peptides with annotated activities, such as the protein associated with Myc (PAM) inhibitor, DPP-III inhibitor, and regulator of phosphoglycerate kinase activity, may also be postulated to exert anticancer effects. Peptides with immunomodulatory and anti-inflammatory properties (including the aminopeptidase inhibitor Xaa-Pro and DPP-III inhibitor) may possess more generalised systemic functions, partially related to cancer prevention and immunity.

One of the most prevalent biological activities of peptides is the inhibition of angiotensin-converting enzyme (ACE), which in silico analyses attributed to all the peptides obtained in this study. This effect involves inhibition of the angiotensin-converting enzyme, which converts angiotensin I (AT I) into angiotensin II (AT II), consequently reducing the blood levels of ATII, which in excess constricts the vessels, resulting in an increase in blood pressure. Previous research has reported that several milk- and whey-derived peptides exhibit high in vitro ACE inhibitory activity (IPP peptides, LIVTQ, IIAE, LVYPFP) [48]. Among the peptides identified in this investigation, APFPEVF was previously predicted to possess ACE inhibitory activity [49].

In addition to hypotensive peptides, blood-pressure-lowering and anti-atherosclerotic effects are also attributed to peptides with calmodulin-dependent cyclic nucleotide phosphodiesterase (CaMPDE) inhibitor, DPP-III inhibitor, and renin inhibitor activity, as well as partially to peptides stimulating vasoactive substance release. Peptides with hypolipidaemic effects are also partially associated with the prevention of cardiovascular disease. This effect was attributed to peptides with HMG-CoA reductase inhibitor activity. In our investigation, in silico analyses indicated that one of the identified peptides (RELKDLKGYGGVS) may exhibit such activity. By inhibiting the HMGR activity, these peptides reduce the rate of cholesterol biosynthesis [50,51].

In contrast to the aforementioned activities, among the predicted activities of the peptides shown in the in silico analyses, anti-diabetic, antiviral, anti-sclerotic, and gut mucosa-regulating activities are considered particularly noteworthy [52]. Several categories of peptide activity are associated with antidiabetic potential and the maintenance of glucose homeostasis. Of the anticipated activities attributed to the peptides identified in this study, peptides with potential DPP-IV inhibitor and alpha-glucosidase inhibitor activities, as well as those stimulating glucagon-like peptide 1 release and regulating phosphoglycerate kinase, may exhibit such effects. Regarding peptides with antiviral activity, the study identified 17 peptides that were found to have aminopeptidase Xaa-Pro inhibitory activity, which may provide an antiretroviral effect. The group of peptides with predicted effects on the nervous system is equally significant. These include peptides with potential neuropeptide, opioid, antiamnestic, and β-casomorphin-5 butyrylcholinesterase inhibitor activities.

## 3. Materials and Methods

### 3.1. Materials

Commercial bovine whey protein isolate (84% protein content) was procured from BioTechUSA Ltd. (Budapest, Hungary). The amino acid composition of the products is presented in Table 3.

### 3.2. Preparation of Whey Hydrolysates

Enzymatic hydrolysis was used to obtain peptides from the whey proteins. Three distinct enzymes were utilised for hydrolysis: pepsin (from porcine gastric mucosa, ≥400 units/mg protein, P7125, Sigma-Aldrich, Saint Louis, MO, USA), papain (from papaya latex crude powder, 1.5–10 units/mg solid, P3375, Sigma-Aldrich, Saint Louis, MO, USA), and trypsin (from bovine pancreas, lyophilised powder, ≥10,000 BAEE units/mg protein, T1426, Sigma-Aldrich, Saint Louis, MO, USA), with the objective of selecting the enzyme that yielded the optimal degree of hydrolysis and facilitated the production of peptides with the highest antioxidant activity. The protein isolate was dissolved in Milli-Q water and preheated, and the pH of the solution was subsequently adjusted to the optimum value for the action of the individual enzymes, namely 3.0, 6.0, and 8.0 for pepsin, papain, and trypsin, respectively. Enzymatic hydrolysis was conducted for 3 h with constant agitation at an enzyme/substrate ratio of 1:10 (*w*/*w*), at 37 °C for pepsin and trypsin, and 70 °C for papain. The reaction was terminated by heating the mixture at 100 °C for 15 min. Subsequently, the hydrolysate was cooled to 37 °C and centrifuged at 3000× *g* for 30 min. The resultant supernatant was lyophilised and stored at 4 °C for further analysis (and the degree of hydrolysis and antioxidant activity were determined for each variant).

### 3.3. Determination of Degree of Hydrolysis

The degree of hydrolysis was determined by quantifying the soluble peptides present in the mixture at predetermined time intervals from the initiation of the hydrolysis process (0, 0.5, 1, 2, and 3 h). For this purpose, 500 µL of the hydrolysate was extracted at specified time points and immediately combined with 10% trichloroacetic acid in a 1:1 (*v*/*v*) ratio. The samples were subsequently incubated for 15 min at room temperature, followed by centrifugation (5500× *g* for 10 min). To ascertain the concentration of soluble peptides in the supernatant, a spectrophotometric method was employed to measure absorbance at 280 nm. The degree of hydrolysis was calculated as the percentage of soluble peptides relative to the total protein content in the mixture.

$$DH\;\left[\%\right]=\frac{protein\;content\;after\;hydrolysis}{total\;protein\;content\;in\;sample}\;\cdot 100\%$$

### 3.4. Determination of Antioxidant Potential

Antioxidant activity of the peptides was determined as previously described by Czelej et al. [53] using 2,20-azino-bis(3-ethylbenzthiazoline-6-sulfonic acid (ABTS•+), 1,1-diphenyl-2-picrylhydrazyl (DPPH), and Ferric Reducing Antioxidant Power (FRAP) assays. Samples were prepared by combining 10 mg of hydrolysate with 1 mL of deionised water (5 min, 15 Hz, Retsch Mixer MM400; Haan, Germany). The samples were centrifuged for 3 min (15 °C, 33 000× *g*, Hettich 32R centrifuge, Tuttlingen, Germany), and the supernatant was used for subsequent analysis. The samples were prepared in triplicates.

The Ferric Reducing Antioxidant Power of the whey peptides was assessed using a 10 mmol/L solution of 2,4,6-Tris(2-pyridyl)-s-triazine (TPTZ) diluted in 40 mmol/L HCl combined with 20 mmol/L FeCl3 · 6H_2_O and acetate buffer (300 mmol/L, pH 3.6) at a 1:1:10 (*v*:*v*:*v*) ratio. Then, 50 µL of the samples containing the peptide solution was combined with 150 µL of FRAP reagent, homogenised, and incubated for 5 min at 25 °C. Subsequently, the absorbance was measured at 593 nm (using a Marcel s330 spectrophotometer, Zielonka, Poland) against a blank sample (containing water instead of the peptide sample). The reducing antioxidant power was assessed by reference to a standard curve established using Trolox standards.

Free radical scavenging activity was evaluated using the stable radical DPPH assay. Aliquots (50 µL) of the peptide solution were combined with 150 µL of methanolic DPPH solution. Samples were incubated in the dark at 25 °C for 60 min with agitation. The absorbance of each sample was measured spectrophotometrically at 517 nm against a blank sample. The antioxidant activity of the peptides was evaluated by comparison with a standard curve plotted against the Trolox standards.

Radical Cation Decolourisation Activity was assayed using (ABTS•+). The ABTS•+ working solution was prepared by combining an equal volume of 7 mM ABTS and 2.4 mM potassium persulfate in deionised water and allowed to stand for 16 h prior to the measurements. The resulting working solution was diluted with phosphate-buffered saline (PBS) until the absorbance reached 0.7 at λ = 734 nm. Then, 50 µL of the working solution was added to 1 mL of the peptide mixture sample, and the absorbance of the samples was measured at 734 nm relative to the blank.

### 3.5. Antibacterial Activity

The antimicrobial activity of the whey peptides was evaluated using a well diffusion assay. This study encompassed eight bacterial strains previously isolated from decomposed fruits and vegetables (*Bacillus* sp., *Bacillus cereus*, *Bacillus megaterium*, *Bacillus pumilus*, *Staphylococcus capitis*, *Exiguobacterium aurantiacum*, *Brevundimonas diminuta*, and *Rhizobium radiobacter*) [53]. Bacterial strains were cultured in Mueller–Hinton broth (Biomaxima, Lublin, Poland) at 37 °C for 24 h. Subsequently, 100 µL of the bacterial culture was applied to Petri dishes containing Mueller–Hinton agar and was distributed uniformly across the surface. For this, 8 mm diameter wells were excised in the medium. Then, 70 µL of the test peptide at a concentration of 50 mg/mL was introduced into the wells. The plates were incubated for 24 h at 37 °C, and the bacterial growth inhibition zones surrounding the wells were measured.

### 3.6. Identification and Characterisation of Bioactive Peptides by LC-MS

For peptide identification, 3 mg of each sample after papain hydrolysis was reconstituted in 400 µL of 100 mM ammonium bicarbonate buffer. The peptides were filtered through a Vivacon 30 kDa molecular weight cutoff filter (Sartorius Stedim Biotech, Goettingen, Germany). The samples were analysed using an LC-MS system comprising an Evosep One HPLC System (Evosep Biosystems, Odense, Denmark) coupled with an Orbitrap Exploris 480 mass spectrometer (Thermo Scientific, Sunnyvale, CA, USA). The peptide solution (100 µL) was loaded onto Evotips Pure C18 disposable trap columns according to the manufacturer’s instructions. The peptides were fractionated for 88 min with a predefined Evosep gradient at a flow rate of 220 nL/min on an analytical column (Dr Maisch C18 AQ, 1.9 µm beads, 150 µm ID, 15 cm long, Evosep Biosystems, Odense, Denmark). The data-dependent acquisition parameters were as follows: top 40 precursors selected for MS2 analysis, collisional induced fragmentation NCE 30%, spray voltage 2.1 kV, funnel RF level 40, and heated capillary temperature 275 °C. Full MS scans covered the mass range of 300–1600 *m*/*z* with a resolution of 60,000, a maximum injection time set to Auto, and a normalised AGC target of 300%. MS2 scans were acquired with a resolution of 15,000, auto maximum injection time, and Standard AGC target. The ion isolation window was set to 1.6 *m*/*z*, dynamic exclusion was set to 20 s, and the minimum intensity threshold was 5 × 10^3^.

Raw data were pre-processed with Mascot Distiller (version 2.8, Matrixscience, London, UK) and peptides/proteins were identified with Mascot suite (version 2.8, Matrixscience) after offline mass recalibration with MScan (version 3.0, http://proteom.ibb.waw.pl/mscan; accessed on 14 February 2023). The search parameters were as follows: enzyme—none, variable modifications—oxidation (M), max missed cleavages—2, fragment ion tolerance—0.01 Da, parent ion tolerance set individually after recalibration within range 7–11 ppm. The database always includes popular contaminants (common Repository of Adventitious Proteins (cRAP), 115 sequences, 86,853,323,495 residues). Species-specific database: Bos taurus (47,245 sequences) from Uniprot.

### 3.7. In Silico Analysis of LC-MS-Identified Peptides

The biological activities of the obtained peptides were analysed based on their amino acid sequences using the BIOPEP-UWM database [54]. Identified peptide sequences were verified for known bioactivities by comparing them against the database’s collection of biologically active peptides. With regard to peptides without a direct match, potential activity was predicted based on their structural and compositional similarities to known bioactive sequences. This approach allowed for a comprehensive assessment of the functional potential of the identified whey-protein-derived peptides.

### 3.8. Statistical Analysis

Statistical analyses were performed using Python 3.10 (SciPy package and statsmodels). One-way ANOVA followed by Tukey’s post hoc test were applied to compare samples treated with different enzymes for each antioxidant parameter (ABTS, FRAP, DPPH). Results are presented as mean ± standard deviation, and *p* values < 0.05 were considered statistically significant.

## 4. Conclusions

In contemporary research, food-derived proteins are increasingly recognised not only for their nutritional value but also as a significant source for identifying bioactive peptide sequences. In this study, the matrix selected was whey, which contains proteins that offer substantial potential for the extraction of bioactive peptides. This study focused on whey, a matrix with considerable potential for extracting bioactive peptides. This article elucidates the hydrolysis of whey proteins and the identification of peptides released during enzymatic reactions, along with their biological activities. These findings demonstrate that high efficiency in the enzymatic hydrolysis process can be achieved by utilising papain at a relatively elevated reaction temperature of 70 °C. Consequently, 107 distinct peptide sequences were obtained and identified using LC-MS.

By employing optimised enzymatic hydrolysis conditions, the majority of the identified peptide sequences represented novel peptides that have not been previously described or recorded in databases. In silico analyses correlated these sequences with a broad spectrum of predicted biological activity. Furthermore, in vitro assays confirmed the antioxidant activity of the whey hydrolysates and their selective antimicrobial properties.

The research presented in this work provides extensive data on novel peptides derived from whey proteins and their potential biological activities. These findings suggest that the hydrolysates obtained could be utilised in the development of functional foods and beverages, dietary supplements, or nutraceuticals. This research establishes a foundation for further investigations to validate the predicted activities observed in in silico and in vitro experiments. Additionally, there is a need to assess their bioavailability and properties in vivo to achieve a comprehensive understanding of their health effects. Future studies should also explore the feasibility of leveraging these peptides for pharmaceutical applications, as well as in functional foods, dietary supplements, and nutraceuticals, where their bioactive properties could offer significant health benefits.

## Figures and Tables

**Table 1:** Antioxidant potential of peptides obtained via enzymatic hydrolysis of whey proteins.

Enzyme Treatment	ABTS (eq Trolox µmol/g)	FRAP (eq Trolox µmol/g)	DPPH (eq Trolox µmol/g)
Pepsin	297.44 ± 24.11 ^a^	16.22 ± 2.03 ^a^	4.87 ± 0.22 ^c^
Trypsin	361.96 ± 31.02 ^b^	19.68 ± 1.5 ^b^	1.75 ± 0.03 ^a^
Papain	527.36 ± 25.13 ^c^	17.56 ± 2.16 ^ab^	3.56 ± 0.17 ^b^

The values followed by different letters in superscripts mark significant differences (*p* < 0.05) for each test obtained by ANOVA analysis and Tukey HSD test.

**Table 2:** Antimicrobial activity of whey peptides hydrolysed with the use of papain.

Bacterial Species	G(+)/G(−)	Growth Inhibition Zone (mm)
*Bacillus* sp.	G(+)	11
*Bacillus cereus*	G(+)	0
*Bacillus megaterium*	G(+)	8
*Bacillus pumilus*	G(+)	0
*Staphylococcus capitis*	G(+)	10
*Exiguobacterium aurantiacum*	G(+)	0
*Brevundimonas diminuta*	G(−)	0
*Rhizobium radiobacter*	G(+)	9

**Table 3:** Amino acid content of WPI used in the study (mg per 100 g WPI).

Essential Amino Acids (mg)		Conditionally Essential Amino Acids (mg)		Nonessential Amino Acids (mg)	
Histidine	1500	Arginine	2008	Alanine	3836
Isoleucine	4712	Cysteine	2552	Aspartic acid	8564
Leucine	8680	Glutamine and glutamic acid	17,208	Glycine	1428
Methionine	1880	Proline	4424	Serine	3804
Phenylalanine	2556	Tyrosine	2384		
Threonine	5176				
Tryptophan	1368				
Valine	4564				

## Data Availability

Data are contained within the article and Supplementary Materials.

## Supplementary Materials

The following supporting information can be downloaded at: https://www.mdpi.com/article/10.3390/molecules30071451/s1, Table S1: The identified peptide sequences derived via papain-catalysed hydrolysis of whey proteins, information on proteins from which they are derived and predicted biological activities of the peptides.

## References

[1] Yu Y.-J., Amorim M., Marques C., Calhau C., Pintado M. (2016). Effects of whey peptide extract on the growth of probiotics and gut microbiota. J. Funct. Foods.

[2] Brandelli A., Daroit D.J., Corrêa A.P.F. (2015). Whey as a source of peptides with remarkable biological activities. Food Res. Int..

[3] Zhao C., Ashaolu T.J. (2020). Bioactivity and safety of whey peptides. LWT—Food Sci. Technol..

[4] Madureira A.R., Pereira C.I., Gomes A.M.P., Pintado M.E., Xavier Malcata F. (2007). Bovine whey proteins—Overview on their main biological properties. Food Res. Int..

[5] Madadlou A., Abbaspourrad A. (2018). Bioactive whey peptide particles: An emerging class of nutraceutical carriers. Crit. Rev. Food Sci. Nutr..

[6] Olvera-Rosales L.B., Cruz-Guerrero A.E., García-Garibay J.M., Gómez-Ruíz L.C., Contreras-López E., Guzmán-Rodríguez F., González-Olivares L.G. (2023). Bioactive peptides of whey: Obtaining, activity, mechanism of action, and further applications. Crit. Rev. Food Sci. Nutr..

[7] Sila A., Bougatef A. (2016). Antioxidant peptides from marine by-products: Isolation, identification and application in food systems. A review. J. Funct. Foods.

[8] Li J., Hartinger C., Zhu F. (2023). Physicochemical properties of infant formula model emulsions stabilised by different whey protein hydrolysates and characteristics of interfacial peptides. Food Hydrocoll..

[9] Saadi S., Saari N., Anwar F., Hamid A.A., Ghazali H.M. (2015). Recent advances in food biopeptides: Production, biological functionalities and therapeutic applications. Biotechnol. Adv..

[10] López E.C., Eberhardt A., Marino F., Mammarella E.J., Sihufe G.A., Manzo R.M. (2022). Physicochemical characterisation of ACE-inhibitory and antioxidant peptides from Alcalase^®^ whey protein hydrolysates using fractionation strategies. Int. J. Dairy Technol..

[11] Halavach T.M., Kurchenko V.P., Tarun E.I., Yantsevich A.V., Shchur V.V., Tsygankow V.G., Lodygin A.D., Evdokimov I.A., Poklar Ulrih N. (2024). Effect of hydrolysis degree with Alcalase on antioxidant and antigenic properties of whey and colostrum protein hydrolysates. J. Agric. Food Res..

[12] Gammoh S., Alu’datt M.H., Alhamad M.N., Alrosan M., Al-husein B., AL-U’datt D.G., Al-kandari S., Rababah T., Ammari Z., Albiss B.A. (2022). Enzymatic bioactive peptides from sonicated whey proteins of camel milk: Impacts of nanopeptides on structural properties, antioxidant activity and inhibitory activity of alpha-amylase and ACE. Int. J. Dairy Technol..

[13] Peslerbes M., Fellenberg A., Jardin J., Deglaire A., Ibáñez R.A. (2022). Manufacture of whey protein hydrolysates using plant enzymes: Effect of processing conditions and simulated gastrointestinal digestion on angiotensin-I-converting enzyme (ACE) inhibitory activity. Foods.

[14] Kankanamge R., Jeewanthi C., Paik H.-D., Kim M.-H., Lee N.-K., Kim S.-Y., Yoon Y.C. (2014). Characteristics of whey protein hydrolysates from cheese whey, favors on various food application. Chem. Ind. Chem. Eng. Q..

[15] Kankanamge R., Jeewanthi C., Kim M.-H., Lee N.-K., Yoon Y.C., Paik H.-D. (2017). Peptide analysis and the bioactivity of whey protein hydrolysates from cheese whey with several enzymes. Korean J. Food Sci. Anim. Resour..

[16] Halavach T.M., Kurchenko V.P., Zhygankov V.G., Evdokimov I.A. (2015). Determination of physicochemical, immunochemical and antioxidant properties, toxicological and hygienic assessment of whey protein concentrate and its hydrolysate. Foods Raw Mater..

[17] Jia C.L., Hussain N., Joy Ujiroghene O., Pang X.Y., Zhang S.W., Lu J., Liu L., Lv J.P. (2020). Generation and characterization of dipeptidyl peptidase-IV inhibitory peptides from trypsin-hydrolyzed α-lactalbumin-rich whey proteins. Food Chem..

[18] Chatterjee A., Kanawjia S.K., Khetra Y., Saini P. (2015). Discordance between in silico & in vitro analyses of ACE inhibitory & antioxidative peptides from mixed milk tryptic whey protein hydrolysate. J. Food Sci. Technol..

[19] Wang R., He S., Xuan Y., Cheng C. (2020). Preparation and characterization of whey protein hydrolisates-Zn complexes. J. Food Meas. Charact..

[20] Giromini C., Lovegrove J.A., Givens D.I., Rebucci R., Pinotti L., Maffioli E., Tedeschi G., Sundaram T.S., Baldi A. (2019). In vitro-digested milk proteins: Evaluation of angiotensin-1-converting enzyme inhibitory and antioxidant activities, peptidomic profile, and mucin gene expression in HT29-mtx cells. J. Dairy Sci..

[21] Ballatore M.B., del Rosario Bettiol M., Braber N.L.V., Aminahuel C.A., Rossi Y.E., Petroselli G., Erra-Balsells R., Cavaglieri L.R., Montenegro M.A. (2020). Antioxidant and cytoprotective effect of peptides produced by hydrolysis of whey protein concentrate with trypsin. Food Chem..

[22] Estévez N., Fuciños P., Fuciños C., Jauregi P., Tovar C.A., Rúa M.L. (2020). Hydrolysis of whey protein as a useful approach to obtain bioactive peptides and a β-lg fraction with different biotechnological applications. Food Hydrocoll..

[23] Jeewanthi R.K.C., Lee N.-K., Paik H.-D. (2015). Improved functional characteristics of whey protein hydrolysates in food industry. Korean J. Food Sci. Anim. Resour..

[24] West C., Gallagher D. (2007). Whey protein—A source of digestible nutrients but palatability issues remain. Food Sci. Technol..

[25] Melnikova E.I., Bogdanova E.V., Koshevarova I.B. (2022). Nutritional evaluation of whey protein hydrolysate: Chemical composition, peptide profile, and osmolarity. Food Sci. Technol..

[26] Zapata Bustamante S., Gil González J., Sforza S., Tedeschi T. (2021). Bioactivity and peptide profile of whey protein hydrolysates obtained from Colombian double-cream cheese production and their products after gastrointestinal digestion. Food Sci. Technol..

[27] Kaur S., Vasiljevic T., Huppertz T. (2023). Milk Protein Hydrolysis by Actinidin—Kinetic and Thermodynamic Characterisation and Comparison to Bromelain and Papain. Foods.

[28] Kordala N., Bednarska M., Adamczak M. (2021). Health-promoting properties of bioactive peptides (BAP) in dairy products—Biotechnological and medical aspects. Med. Og. Nauk. Zdr..

[29] Du X., Jing H., Wang L., Huang X., Wang X., Wang H. (2022). Characterization of structure, physicochemical properties, and hypoglycemic activity of goat milk whey protein hydrolysate processed with different proteases. LWT—Food Sci. Technol..

[30] Kumar D., Chatli M.K., Singh S., Mehta N., Kumar P. (2016). Antioxidant and antimicrobial activity of camel milk casein hydrolysates and its fractions. Small Rumin. Res..

[31] Wu H.C., Chen H.M., Shiau C.Y. (2003). Free amino acids and peptides as related to antioxidant properties in protein hydrolysates of mackerel (*Scomber austriasicus*). Food Res. Int..

[32] Matsui R., Honda R., Kanome M., Hagiwara A., Matsuda Y., Togitani T., Ikemoto N., Terashima M. (2018). Designing antioxidant peptides based on the antioxidant properties of the amino acid side-chains. Food Chem..

[33] Ohashi Y., Onuma R., Naganuma T., Ogawa T., Naude R., Nokihara K., Muramoto K. (2015). Antioxidant properties of tripeptides revealed by a comparison of six different assays. Food Sci. Technol. Res..

[34] Martysiak-Żurowska D., Wenta W. (2012). A comparison of ABTS and DPPH methods for assessing the total antioxidant capacity of human milk. Acta scientiarum polonorum. Acta Sci. Pol. Technol. Aliment..

[35] Chen J., Lindmark-Månsson H., Gorton L., Åkesson B. (2003). Antioxidant capacity of bovine milk as assayed by spectrophotometric and amperometric methods. Int. Dairy. J..

[36] Thwaite J.E., Hibbs S., Titball R.W., Atkins T.P. (2006). Proteolytic Degradation of Human Antimicrobial Peptide LL-37 by Bacillus anthracis May Contribute to Virulence. Antimicrob. Agents.

[37] Abdel-Hamid M., Goda H.A., De Gobba C., Jenssen H., Osman A. (2016). Antibacterial activity of papain hydrolyzed camel whey and its fractions. Int. Dairy J..

[38] Salami M., Moosavi-Movahedi A.A., Ehsani M.R., Yousefi R., Haertlé T., Chobert J.M., Razavi S.H., Henrich R., Balalaie S., Ebadi S.A. (2010). Improvement of the antimicrobial and antioxidant activities of camel and bovine whey proteins by limited proteolysis. J. Agric. Food Chem..

[39] Meignanalakshmi S., Vinoth Kumar S. (2013). Antibacterial activity of papain hydrolysates of buffalo milk whey milk whey protein against mastitis pathogens. Int. J. Pharma Bio Sci..

[40] Pellegrini A., Dettling C., Thomas U., Hunziker P. (2001). Isolation and characterization of four bactericidal domains in the bovine β-lactoglobulin. BBA—Gen. Subj..

[41] Fu T., Zhang S., Sheng Y., Feng Y., Jiang Y., Zhang Y., Yu M., Wang C. (2020). Isolation and characterization of zinc-binding peptides from mung bean protein hydrolysates. Eur. Food Res. Technol..

[42] Udechukwu M.C., Dang C., Udenigwe C.C. (2021). Identification of zinc-binding peptides in ADAM17-inhibiting whey protein hydrolysates using IMAC-Zn^2+^ coupled with shotgun peptidomics. Food Prod. Process Nutr..

[43] Maugard T., Bordenave-Juchereau S., Piot J.M., Ben-Henda Y., Sirvent P., Peltier S. (2018). Compositions for Preventing and/or Treating Pathological Conditions Associated with Alpha-Glucosidase. US.

[44] Peltier S., Sirvent P., Maugard T. (2018). Compositions and Methods for Treating Diabetes, Fatty Liver, Cardiopathies, Insulin Resistance, Carbohydrate and Fat Me-tabolism. US.

[45] Hsieh C.-H., Wang T.-Y., Hung C.-C., Hsieh Y.-L., Hsu K.-C. (2016). Isolation of prolyl endopeptidase inhibitory peptides from a sodium caseinate hydrolysate. Food Funct..

[46] Meisel H., Frister H. (1989). Chemical characterization of bioactive peptides from in vivo digests of casein. J. Dairy Res..

[47] de Vasconcelos M.L., Oliveira L.M.F.S., Hill J.P., Vidal A.M.C. (2023). Difficulties in Establishing the Adverse Effects of β-Casomorphin-7 Released from β-Casein Variants—A Review. Foods.

[48] Chamata Y., Watson K.A., Jauregi P. (2020). Whey-Derived Peptides Interactions with ACE by Molecular Docking as a Potential Predictive Tool of Natural ACE Inhibitors. Int. J. Mol. Sci..

[49] FitzGerald R.J., Cermeño M., Khalesi M., Kleekayai T., Amigo-Benavent M. (2020). Application of in silico approaches for the generation of milk protein-derived bioactive peptides. J. Funct. Foods.

[50] Bansal A.B., Cassagnol M. (2024). HMG-CoA Reductase Inhibitors. StatPearls.

[51] Pak V.V., Koo M., Kwon D.Y., Yun L. (2012). Design of a highly potent inhibitory peptide acting as a competitive inhibitor of HMG-CoA reductase. Amino Acids.

[52] Maestri E., Pavlicevic M., Montorsi M., Marmiroli N. (2019). Meta-Analysis for Correlating Structure of Bioactive Peptides in Foods of Animal Origin with Regard to Effect and Stability. Compr. Rev. Food Sci. Food Saf..

[53] Czelej M., Czernecki T., Garbacz K., Wawrzykowski J., Jamioł M., Michalak K., Walczak N., Wilk A., Waśko A. (2023). Egg Yolk as a New Source of Peptides with Antioxidant and Antimicrobial Properties. Foods.

[54] Minkiewicz P., Iwaniak A., Darewicz M. (2019). BIOPEP-UWM Database of Bioactive Peptides: Current Opportunities. Int. J. Mol. Sci..

